# The association of energy and macronutrient intake with all-cause mortality, cardiovascular disease and dementia: findings from 120 963 women and men in the UK Biobank

**DOI:** 10.1017/S000711452100266X

**Published:** 2022-06-28

**Authors:** Briar L. McKenzie, Katie Harris, Sanne A. E. Peters, Jacqui Webster, Mark Woodward

**Affiliations:** 1 The George Institute for Global Health, University of New South Wales, Sydney, Australia; 2 The George Institute for Global Health, Imperial College, London, UK; 3 Julius Center for Health Sciences and Primary Care, University Medical Center Utrecht, Utrecht, The Netherlands; 4 Welch Center for Prevention, Epidemiology and Clinical Research, Johns Hopkins University, Baltimore, MD, USA

**Keywords:** Dietary intake, Diet assessment, Carbohydrate intake, Fat intake, Protein intake, CVD, Dementia, Sex differences

## Abstract

This study aimed to investigate the association between individual and combinations of macronutrients with premature death, CVD and dementia. Sex differences were investigated. Data were utilised from a prospective cohort of 120 963 individuals (57 % women) within the UK Biobank, who completed ≥ two 24-h diet recalls. The associations of macronutrients, as percentages of total energy intake, with outcomes were investigated. Combinations of macronutrients were defined using k-means cluster analysis, with clusters explored in association with outcomes. There was a higher risk of death with high carbohydrate intake (hazard ratios (HR), 95 % CI upper *v.* lowest third 1·13 (1·03, 1·23)), yet a lower risk with higher intakes of protein (upper *v.* lowest third 0·82 (0·76, 0·89)). There was a lower risk of CVD with moderate intakes (middle *v.* lowest third) of energy and protein (sub distribution HR (SHR), 0·87 (0·79, 0·97) and 0·87 (0·79, 0·96), respectively). There was a lower risk of dementia with moderate energy intake (SHR 0·71 (0·52, 0·96)). Sex differences were identified. The dietary cluster characterised by low carbohydrate, low fat and high protein was associated with a lower risk of death (HR 0·84 (0·76, 0·93)) compared with the reference cluster and a lower risk of CVD for men (SHR 0·83 (0·71, 0·97)). Given that associations were evident, both as single macronutrients and for combinations with other macronutrients for death, and for CVD in men, we suggest that the biggest benefit from diet-related policy and interventions will be when combinations of macronutrients are targeted.

In the UK, the burden of disease due to poor diets is estimated to be 10 % of the total disease burden^(1)^. Previous studies have identified increased risk of non-communicable disease and mortality with high saturated and trans-fat^(2–4)^, high intake of added sugar^(5)^ and decreased risks with higher protein^(6,7)^ and fibre intake^(4,8)^. The vast majority of these studies were observational, with a focus on CVD. Evidence of the relationship between diet and disease has been used to set dietary recommendations for energy and macronutrients, globally and in the UK^(9)^. These recommended values provide an insight into what a standard diet for a ‘healthy’ individual should be made up of, in terms of energy, carbohydrates, fats and protein. Dietary recommendations generally provide a cut-off for intake of individual macronutrients, for example fat intake should be <35 % of energy intake a day^(9)^. However, given that nutrients are not eaten in isolation and many nutrients interact with each other, it becomes difficult to explore the relationship between the individual dietary recommendations and disease outcomes^(10)^.

In order to implement effective food policies and set relevant guidelines, the risk between poor diets and disease needs to be frequently monitored, particularly in relation to diseases contributing the highest burden. In the UK, the leading cause of death for men is ischaemic heart disease, for women it is dementia^(11)^. While it is acknowledged that diet is associated with CVD, the relationship between diet and dementia is less well established^(12)^. Given there is a vascular component to dementia, it is plausible that disease risk would be influenced by dietary intake in a similar manner to CVD^(12)^. The treatment options for dementia are currently limited; therefore, the identification of modifiable risk factors to prevent dementia is urgently needed.

Participants in the UK Biobank^(13)^ provided data on a range of risk factors, including dietary intake, at baseline and their data are linked to hospital admission data and mortality records. Previously, we utilised these data to explore sex differences in macronutrient intake^(14)^ and identified low compliance to dietary recommendations across the study population, with substantial differences in meeting dietary recommendations between men and women. We hypothesised that such low compliance may relate to health outcomes, with the potential for differing impacts by sex.

Therefore, the aims of the present study were to use the UK Biobank data to investigate (1) the association between individual macronutrients with all-cause mortality, CVD and dementia, (2) the association between combinations of macronutrients with all-cause mortality, CVD and dementia and (3) any sex differences in the associations of individual macronutrients and combinations of macronutrients with these outcomes.

## Methods

### Data source

The UK Biobank^(15)^ contains information on over half a million women and men, aged 40–69 years at baseline. Participants volunteered to join the study and completed baseline assessment between 2006 and 2010. Assessments were carried out across twenty-two research centres in the UK and involved the collection of self-reported (questionnaire) data, physical measurements and biological samples.

This research has been conducted using the UK Biobank Resource (application no. 2495). Permission to use the UK Biobank Resource was approved by the access subcommittee of the UK Biobank Board. All participants provided electronic informed consent. The UK Biobank has obtained Research Tissue Bank approval from its governing research ethics committee, as recommended by the National Research Ethics Service. Additional ethical approval for the present study was gained via the University of New South Wales (HC 20056). The study was conducted in accordance with the principles of the Declaration of Helsinki.

### Dietary measures

A web-based 24-h dietary assessment, ‘Oxford WebQ’^(16,17)^, was introduced into the UK Biobank study protocol in 2009. The assessment includes questions on the consumption of 206 types of food and thirty-two types of drinks and asks about consumption in the previous 24 h. Participants who completed their baseline assessment during the last year of recruitment completed the 24-h diet recall survey at the assessment centre. All other participants who provided an email address were invited to complete the 24-h diet recall survey online, at four points ranging between 2 and 6 years post baseline data collection. Nutrients from the surveys were calculated based on the frequency, standard portion size and nutrient composition of the food selected^(15,16)^. For the present study, the nutrients of interest were total energy intake, fat intake (total, saturated, polyunsaturated), carbohydrate intake (total, sugar, fibre) and protein intake. In order to estimate habitual energy intake, two or more 24-h diet recalls are required^(18)^. It was hypothesised that people who have an event (for example, a cardiovascular event or a dementia diagnosis) may change their diet post event^(19)^. As such, only individuals with two or more 24-h diet recalls, without an event occurring between measures, were included in this study and the average of their dietary intake values were calculated. Energy intakes more than four sd from the mean were considered implausible, and individuals with these extreme measures were excluded from analyses (*n* 1034)^(20)^.

### Outcome measures

Outcomes analysed were all-cause mortality (death), fatal or non-fatal CVD and fatal or non-fatal dementia recorded up until 30 June 2020. Mortality and cause of death were identified through linkage to the Office for National Statistics mortality records. Non-fatal CVD and dementia events were determined through linkage to Hospital Episode Statistics for England, Scottish Morbidity Record data for Scotland and the Patient Episode Database for Wales. Diagnoses were recorded using the International Classification of Diseases-10 coding system, with CVD defined using I60, I61, I63, I64, I21, I22, I23, I241, I252, comprising stroke (I60, I61, I63, I64) and myocardial infarction (I21, I22, I23, I241, I252). Dementia was comprised of International Classification of Diseases-10 codes A81·0, F00, F01, F02, F03, F05, G30, G31·0, G31·1, G31·8 and I67·3, with subtype Alzheimer’s disease (F00, G30); and vascular dementia (F01, I67·3). Individuals with a self-reported history of CVD or dementia diagnosis at baseline were excluded from all analyses.

### Statistical analysis

Dietary intake was assessed as mean energy intake and macronutrients as a percentage of total energy intake (carbohydrate, sugar, fibre, fat, saturated fat, polyunsaturated fat and protein) and split into thirds whereby the lowest intake third was the reference (online Supplementary Table 1). The percentage of the population not meeting dietary recommendations was also assessed, in relation to the UK dietary recommendations^(9,14)^. In order to define prevalent combinations of macronutrient intakes, cluster analysis was undertaken using the k-means method, which partitions individuals into clusters such that individuals in the same cluster are as similar as possible^(21)^. Individuals were clustered based on the percentage of total energy intake of carbohydrate, sugar, fibre, total fat, saturated fat, polyunsaturated fat and protein. Clusters were standardised, and naming and definition of clusters were based on the standardised values, with a >0·5 difference from 0 used to name the clusters as ‘high’ or ‘low’ in a certain macronutrient. The largest cluster was used as the reference in the models. Further details on the approach to identifying and defining the clusters are provided in the supplementary material.

For baseline characteristics, categorical variables are presented as number (percentage) and continuous variables are presented as means (standard deviation). Cox proportional hazard models were used to estimate hazard ratios (HR) and 95 % confidence intervals (CI) for all-cause mortality, CVD and dementia. Dietary intake as an exposure of interest was investigated in three forms: (1) absolute intake of energy and percentage energy intake of macronutrients (in thirds), (2) not meeting *v*. meeting dietary recommendations and (3) combinations of macronutrients (standardised cluster variables). Dietary variables were inputted into separate Cox models for each outcome of interest. For the population as a whole, base models were adjusted for age, smoking status, sex and socio-economic status (Townsend deprivation index). Final (multivariable) models were further adjusted for height, weight, physical activity (total metabolic equivalents (MET)), mean alcohol intake, systolic blood pressure, diabetes, lipid-lowering medication and anti-hypertensive medication. Competing risk analyses, producing sub-distribution HR, were conducted for CVD and dementia accounting for all-cause mortality as a competing risk, since death may preclude CVD and dementia from occurring^(22)^. A sensitivity analysis was conducted, looking at different types of dementia (Alzheimer’s disease and vascular dementia, separately). Analyses were also conducted investigating sex interactions with confounders and exposures of interest. For these analyses, the same confounders were used within models with the exception of sex, which was used as an interaction term instead of a confounding variable. Analyses were performed using Stata version 16.0 (StataCorp, 2019) and R Studio Version 4.0.2 (R Core Team, 2020).

## Results

### Characteristics

Twenty percent of the UK Biobank population completed two or more 24-h diet recalls (*n* 120 963; 57 % women). Of these, 45 770 people completed two measures, 40 567 completed three measures, 29 106 completed four measures and 5520 completed diet recalls in all five surveys. Their mean age was 56 years (55·5 years for women and 56·5 years for men) at baseline, and 60 % (53 % of women and 69 % of men) were classified as overweight or obese (BMI ≥ 25 kg/m^2^), Table 1. During a mean of 11·1 years follow-up, there were 2616 cardiovascular events (36.9% women), 292 dementia diagnoses (48.3% women) and 4040 deaths (45.0% women).

**Table 1.** Summary characteristics of participants with two or more dietary assessment measures, by sex

	Overall		Women		Men	
Characteristics	*n*	%	*n*	%	*n*	%
*n*	120 963		68 927		52 036	
Age, years						
Mean	55·9		55·5		56·5	
sd	7·8		7·7		7·9	
Ethnicity, white	116 755	96·9	66 510	96·8	50 245	97·0
Socio-economic status quintiles (Townsend score)						
1st Least deprived	48 335	40·0	26 880	39·0	21 455	41·3
2nd	25 551	21·1	14 728	21·4	10 823	20·8
3rd	18 511	15·3	10 693	15·5	7818	15·0
4th	15 664	13·0	9214	13·4	6450	12·4
5th Most deprived	12 760	10·6	7330	10·6	5430	10·4
Smoking status, never smoked	69 940	57·9	42 195	61·3	27 745	53·4
BMI, kg/m^2^						
Mean	26·6		26·2		27·2	
sd	4·6		4·9		4·0	
Overweight or obese	71 985	59·6	36 109	52·5	35 876	69·1
Weight, kg						
Mean	76·6		70·2		85·0	
sd	15·5		13·6		13·7	
Height, cm						
Mean	169·3		163·6		176·8	
sd	9·2		6·2		6·7	
Low physical activity*	21 641	18·9	12 194	18·8	9447	18·9
Systolic blood pressure (mmHg)	136·6	18·2	133·7	18·7	140·4	16·9
Diastolic blood pressure (mmHg)	81·8	10·0	80·2	9·8	83·9	9·7
Blood pressure categories†						
Normal	19 978	16·5	15 505	22·5	4473	8·6
Elevated	16 023	13·3	9709	14·1	6314	12·1
Stage 1 hypertension	33 372	27·6	18 559	27·0	14 813	28·5
Stage 2 hypertension	51 499	42·6	25 082	36·4	26 417	50·8
Diabetes	4092	3·4	1693	2·5	2399	4·6
Lipid-lowering medication	11 598	9·6	4507	6·5	7091	13·6
Anti-hypertensive medication	14 072	11·6	6559	9·5	7513	14·4
Dietary macronutrient intakes						
	Mean	sd	Mean	sd	Mean	sd
Energy (kJ)	8819	2237	8241	1981	9586	2322
Fats (g)						
Total fat (g)	78·2	26·2	73·5	23·7	84·4	27·8
% EI	32·5	5·8	32·7	5·8	32·3	5·8
Saturated fat (g)	30·0	11·4	28·1	10·3	32·5	12·1
% EI	12·5	2·9	12·5	2·9	12·4	3·0
Polyunsaturated fat (g)	14·4	6·1	13·6	5·7	15·4	6·5
% EI	6·0	1·9	6·1	1·9	5·9	1·9
Carbohydrates (g)						
Total carbohydrate (g)	253·2	74·5	239·2	69·2	271·8	77·2
% EI	48·9	7·6	49·3	7·5	48·3	7·7
Total sugar (g)	119·9	42·5	115·8	41·5	125·3	45·4
% EI	23·2	6·4	23·9	6·4	22·3	6·2
Fibre (g)	16·4	5·8	16·2	5·6	16·7	6·0
% EI	1·5	0·5	1·6	0·5	1·4	0·5
Total protein (g)	82·2	21·3	78·4	19·5	87·1	22·5
% EI	16·1	3·1	16·4	3·2	15·6	2·9

Values are as at baseline except for dietary variables that are averaged over all recorded values. Some variables may not sum to the overall numbers due to missingness.

* Low physical activity defined as < 600 total MET a week.

† Blood pressure categories calculated using the American Heart Association’s 2017 Hypertension Clinical Guidelines^(41)^. Normal – SBP < 120 mmHg and DBP < 80 mmHg, elevated – SBP 120–129 mmHg and DBP < 80 mmHg, stage 1 hypertension SBP 130–139 mmHg or DBP 80–89 mmHg, stage 2 hypertension SBP ≥ 140 mmHg or DBP ≥ 90 mmHg. EI, energy intake.

Compared with the population who did not complete two or more 24-h diet recalls, this population was more likely to live in the least deprived areas, to have never smoked, and a lower percentage had overweight or obesity (online Supplementary Table 2).

### Dietary intake

Mean absolute intakes of energy and macronutrients were higher for men than women. However, as a percentage of energy intake, macronutrient intakes were higher for women (Table 1). When looking at macronutrient intake in terms of dietary recommendations, 38 % of the population exceeded energy intake recommendations. For carbohydrates, 55 % did not meet recommendations, while 63 % exceeded sugar intake recommendations, and the vast majority (98 %) did not meet fibre recommendations. For fat intake, a third of the population (33 %) exceeded recommendations, with 69 % exceeding saturated fat and 55 % not meeting polyunsaturated fat recommendations. Twelve percent of the population did not meet protein recommendations (online Supplementary Table 3).

### Association of individual macronutrient intakes with outcomes

**Fig. 1.**
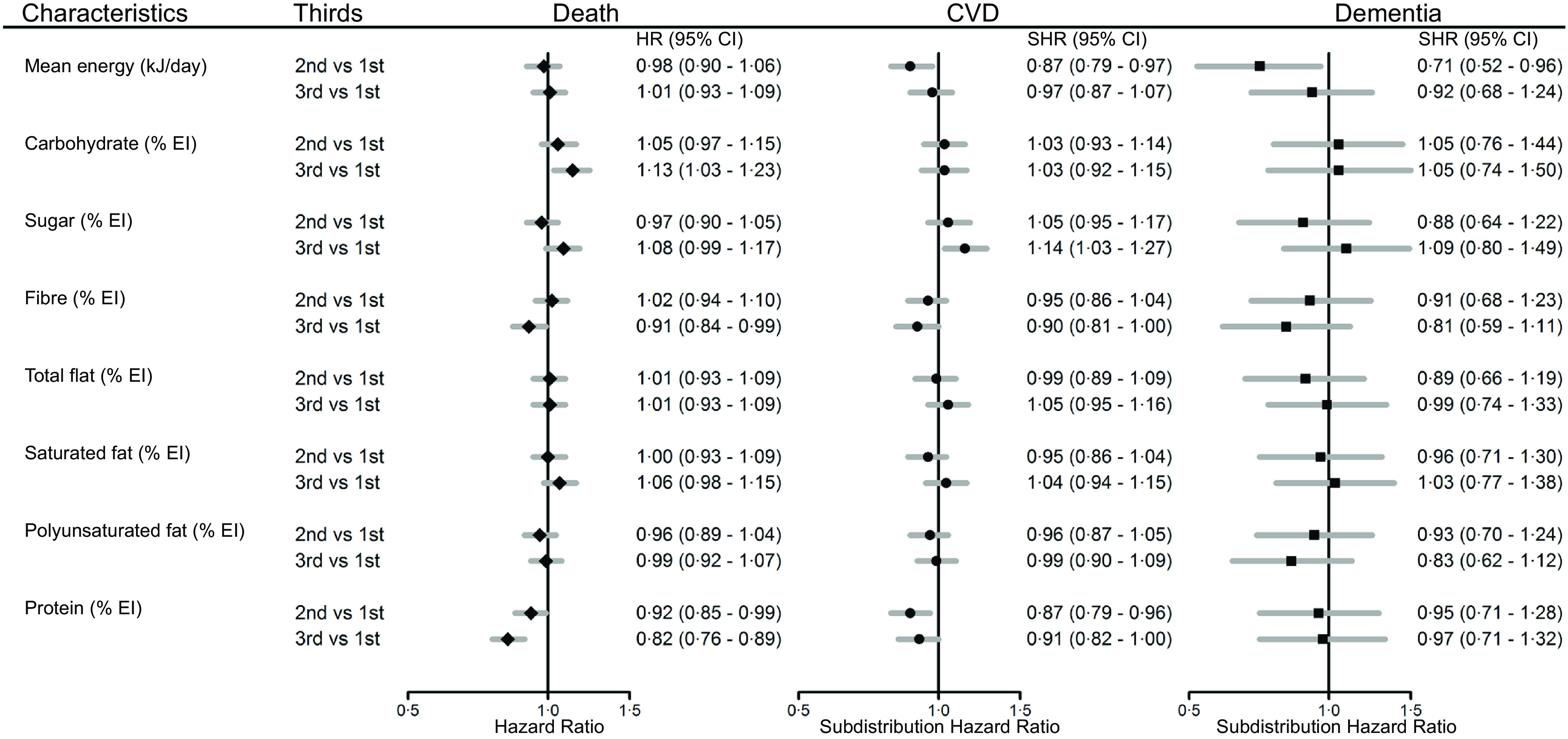
Macronutrient intake (as a percentage of total energy intake, in thirds) and multiple adjusted hazard ratios (HR) for all-cause mortality, subdistribution hazard ratios (SHR) for cardiovascular disease (CVD) and dementia, with 95 % confidence intervals (CI). Models adjusted for age, smoking, sex, height, weight, mean alcohol intake, physical activity (mean total MET), systolic blood pressure, Townsend score, diabetes, lipid-lowering medication, antihypertensive medication (*n* 114 102).

In the multivariable models, only carbohydrate and protein intake were associated with all-cause mortality (Fig. 1). The highest third of total carbohydrate intake (as a percentage of total energy intake) was associated with a higher risk of death, compared with the lowest third (HR 1·13, 95 % CI 1·03, 1·23). A higher percentage energy intake of protein was associated with a lower risk of death (HR middle *v.* lowest third of intake 0·92, 95 % CI 0·85, 0·99, highest *v.* lowest third 0·82, 95 % CI 0·76, 0·89). Individuals with the middle third of mean energy intake had a lower risk of CVD (standardised hazard ratio (SHR) middle *v.* lowest third 0·87, 95 % CI 0·78, 0·96), individuals with high sugar intake had a higher risk of CVD (SHR highest *v.* lowest third of intake, 1·14, 95 % CI 1·03, 1·27) and individuals with the middle third of protein intake had a lower risk of CVD (SHR middle *v.* lowest third 0·87 95 % CI 0·79, 0·96). For dementia, individuals with the middle third of mean energy intake had a lower risk (SHR middle *v.* lowest third 0·71, 95 % CI 0·52, 0·96). Results from the models adjusted for age, smoking, sex and deprivation are reflective of the findings from the multivariable adjusted model (online Supplementary Fig. 1). Additionally, for dementia, we investigated the association between energy and macronutrient intake for Alzheimer’s disease and vascular dementia, separately, finding no association between variables of interest and outcomes (online Supplementary Table 4).

Sex differences in associations between dietary intake and health outcomes were identified, such that, for women those with the highest third of carbohydrate intake had a higher risk of death (HR highest *v.* lowest third of intake, 1·17, 95 % CI 1·02, 1·34), whereas for men, those with the highest third of sugar intake had a higher risk of death (HR highest *v.* lowest third of intake, 1·17, 95 % CI 1·05, 1·31). Women with the highest third of sugar intake had a lower relative risk of death compared with men (ratio of hazard ratios (RHR), women to men, highest *v.* lowest third of intake, 0·81, 95 % CI 0·69, 0·96). Conversely, relative to men, women with moderate total fat intake had a higher risk of death (RHR, women to men, middle *v.* lowest third of intake, 1·20, 95 % CI 1·03, 1·41), Supplementary Table 5. For CVD, men with a moderate energy intake had a lower risk (SHR middle *v.* lowest third of intake, 0·83, 95 % CI 0·72, 0·95). Men with moderate and high protein intakes also had a lower risk of CVD (HR middle *v.* lowest third of intake, 0·87, 95 % CI 0·77, 0·98, highest *v.* lowest third of intake, 0·87, 95 % CI 0·77, 0·99). These associations were not identified for women, Supplementary Table 6. For dementia, moderate sugar intake was associated with a lower risk in women (HR middle *v.* lowest third, 0·51, 95 % CI 0·30, 0·85), and a lower relative risk compared with men (RHR women compared with men, middle *v.* lowest third of intake 0·40, 95 % CI 0·21, 0·77). Additionally, women with the highest third of fibre intake had a lower risk of dementia (HR highest *v.* lowest third of intake, 0·57, 95 % CI 0·37, 0·88) and a lower relative risk compared with men (RHR women compared with men, highest *v.* lowest third of intake 0·52, 95 % CI 0·28, 0·96). Conversely, women with the highest third of saturated fat intake had a higher risk of dementia (HR highest *v.* lowest third of intake 1·69, 95 % CI 1·06, 2·68) and a higher risk relative to men (RHR women compared with men, highest *v.* lowest third of intake 2·49, 95 % CI 1·33, 4·63), Supplementary Table 7.

Models investigating the association of compliance to the individual dietary recommendations (not meeting compared with meeting recommendations) produced similar results to the individual macronutrient analysis (online Supplementary Table 8).

### Cluster analysis

**Fig. 2.**
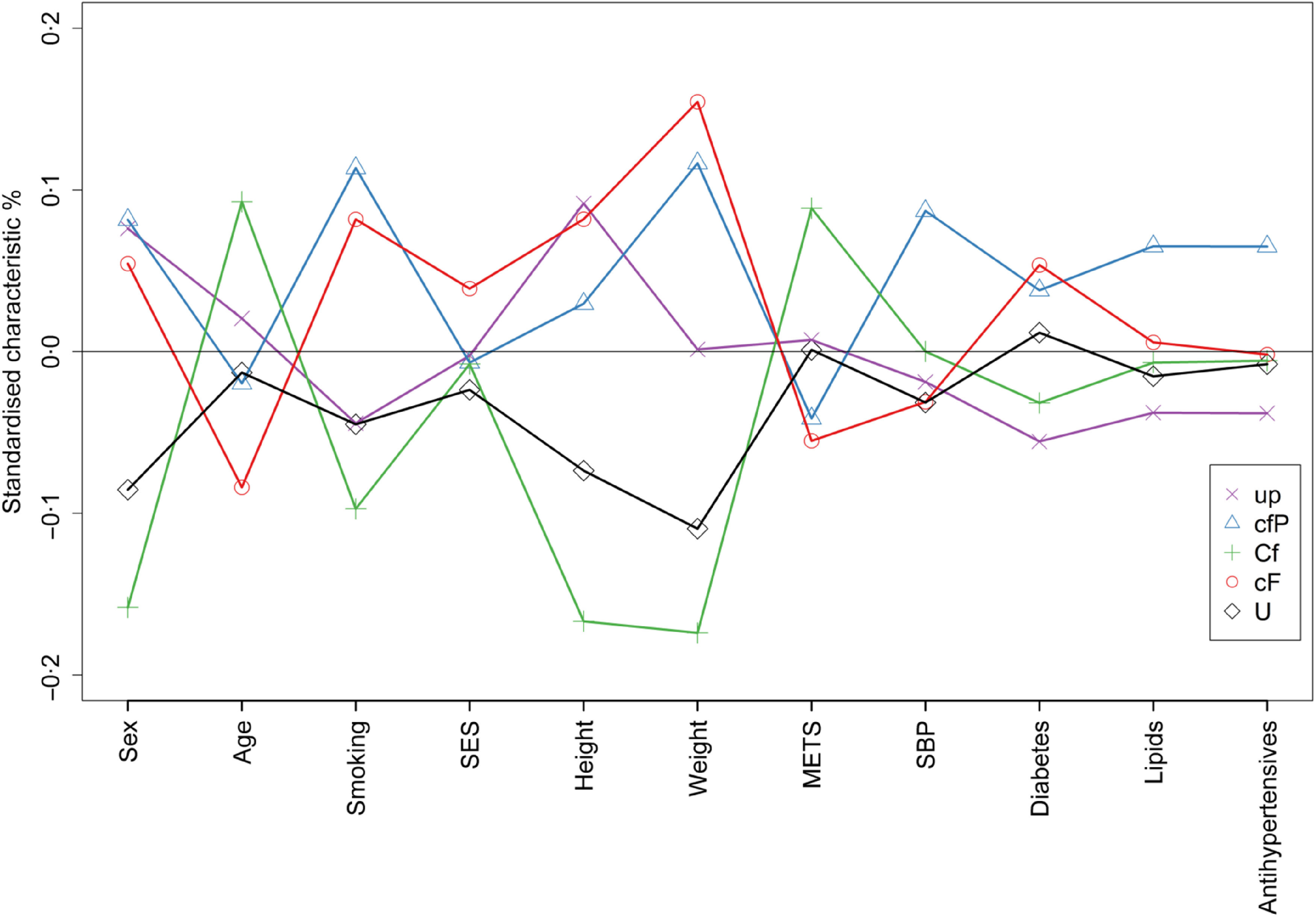
Characteristics of individuals within the identified macronutrient clusters. Macronutrient clusters: *up* – low polyunsaturated fat, low protein, *cfP –* low carbohydrate, low fat, high protein, *Cf* – high carbohydrate, low fat, *cF* – low carbohydrate and high fat, *U*– high polyunsaturated fat. Characteristics: SES - socioeconmic status measured by Townsend score, METS - metabolic equivalents, SBP - systolic blood pressure, Lipids - lipid lowering medication. For each characteristic on the graph, negative points imply a higher proportion of women, younger age, a higher proportion having never smoked, higher proportion living with a higher deprivation level, lower height and weight, lower METs, lower SBP, lower proportion with diabetes, lower proportion on lipid lowering medication and lower proportion on antihypertensive medication compared to the study population mean.

Cluster analysis identified five distinct dietary clusters (Table 2, online Supplementary Fig. 2); low polyunsaturated fat and low protein intake (*up, n* 30 231), low carbohydrate, low fat and high protein intake (*cfP, n* 22 700), high carbohydrate and low fat intake (*Cf, n* 22 215), low carbohydrate and high fat intake (*cF, n* 23 668) and high polyunsaturated fat intake (*U, n* 22 149). Socio-demographic characteristics differed by cluster (Fig. 2, online Supplementary Table 9), a higher proportion of people within the *up* and *cfP* dietary clusters were men (46·7 % and 47·0 %, respectively), whereas a higher proportion of people within the *Cf* and *U* were women (64·7 % and 61·2 %, respectively), compared with the study mean (43 % male). A higher proportion of people within the *Cf* dietary cluster had never smoked (62·6 %, compared with the study mean, 57·9 %).

**Table 2.** Dietary characteristics of clusters; macronutrients shown as a percentage of total energy intake

Cluster	Cluster size	Energy (kJ/d)*	Carbohydrate (CHO)	Sugar	Fibre	Fat	Saturated fat (SFA)	Polyunsaturated fat (PUFA)	Protein
*up –* low PUFA, low protein†	30 231	9460·86	52·19	25·96	1·32	32·31	13·66	5·03	14·30
*cfP –* low CHO low fat, high protein	22 700	8173·76	45·06	20·23	1·41	29·47	11·11	5·27	18·61
*Cf* – high CHO, low fat	22 215	7950·70	57·47	30·33	2·01	25·80	9·52	4·96	16·39
*cF* – low CHO, and high fat	23 668	9245·26	40·98	17·52	1·24	39·45	15·77	6·66	16·27
*U* – high PUFA	22 149	9023·98	48·15	21·53	1·72	35·34	11·62	8·37	15·48
Clusters, standardised‡									
* up*	30 231	NA	0·43	0·43	–0·40	–0·04	0·41	–0·51	–0·58
* cfP*	22 700	NA	–0·50	–0·47	–0·22	–0·53	–0·46	–0·38	0·82
* Cf*	22 215	NA	1·13	1·11	0·98	–1·16	–1·00	–0·54	0·10
* cF*	23 668	NA	–1·04	–0·89	–0·57	1·19	1·13	0·35	0·06
* U*	22 149	NA	–0·10	–0·26	0·40	0·48	–0·28	1·25	–0·20

* The mean energy intake per cluster is shown here for reference; since macronutrients were included as a percentage of total energy intake, mean energy intake was not used to determine the dietary clusters.

† Naming and definition of clusters were based on the standardised values, with a >0·5 difference from 0 used to name the clusters as ‘high’ or ‘low’ in certain macronutrients.

‡ Clusters were standardised to aid comparison, and the standardised clusters were then used in analyses.

### Association of combinations of macronutrients with outcomes

**Fig. 3.**
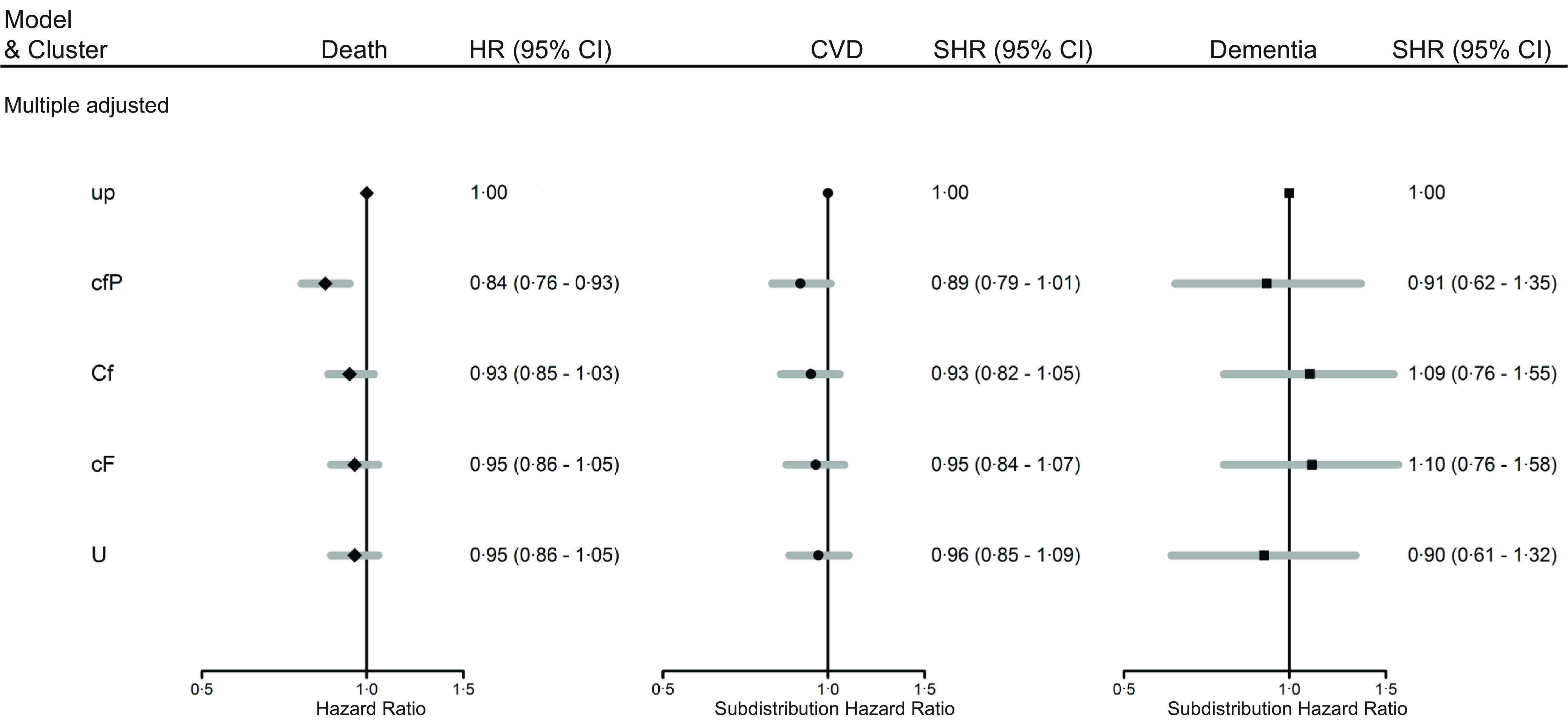
Hazard ratios (HR) for outcomes of all-cause mortality (death), and subdistribution hazard ratios (SHR) for CVD and dementia with 95 % CI, from models adjusted for models adjusted for clusters, age, sex, smoking, height, weight, mean alcohol intake, physical activity (mean total MET), systolic blood pressure, Townsend score, diabetes, lipid-lowering medication, antihypertensive medication (*n* 114 102). Macronutrient clusters: *up* – low polyunsaturated fat, low protein, *cfP –* low carbohydrate, low fat, high protein, *Cf* – high carbohydrate, low fat, *cF* – low carbohydrate and high fat, *U*– high polyunsaturated fat.

From the models for the population as a whole, with multiple adjustments (Fig. 3), the *cfP* dietary cluster was associated with a lower risk of all-cause mortality compared with the *up* (reference) dietary cluster (HR *cfP v. up* 0·84, 95 % CI 0·76, 0·93). There were no associations identified between the dietary clusters with the risk of CVD or dementia. Results from the models adjusted for age, smoking, sex and deprivation are reflective of the findings from the multivariable adjusted model (online Supplementary Fig. 3).

From the sex-specific models with multiple adjustments, the *cfP* cluster was associated with a lower risk of all-cause mortality for women and men (HR *cfP v*. *up* cluster 0·82, 95 % CI 0·70, 0·96 and 0·86, 95 % CI 0·75, 0·98, women and men, respectively), Supplementary Table 10. Men within the *cfP* cluster had a lower risk of CVD (SHR *cfP v*. *up* cluster 0·83, 95 % CI 0·71, 0·97), Supplementary Table 11. From the model with basic adjustments, relative to men, women within the *U* dietary cluster had a lower relative risk of dementia (ratio of SHR’s women to men, *U v*. *up* cluster, 0·40, 95 % CI 0·19, 0·84). This association was attenuated in the model with multiple adjustments (ratio of SHR’s women to men, *U v*. *up* cluster, 0·49, 95 % CI 0·23, 1·05), Supplementary Table 12.

## Discussion

In this large prospective study of over 100 000 women and men from the UK Biobank, associations between energy and macronutrient intakes with all-cause mortality, CVD and dementia were evaluated. We identified a range of associations between individual macronutrients with outcomes. Higher absolute intakes of protein were associated with a decreased risk of all-cause mortality, yet high intakes of carbohydrate were associated with an increased risk of premature death. For CVD and dementia, moderate total energy intake was associated with a decreased risk. Higher sugar intake was associated with an increased risk of CVD, whereas moderate protein intake was associated with a decreased risk. When looking at combinations of dietary factors, individuals with diets characterised by low carbohydrate, low fat and high protein intake had a lower risk of all-cause mortality. Sex differences were identified in the associations between individual macronutrients and outcomes; however, these differences were only reflected in the multiple adjusted cluster analyses for men, where men with diets characterised by low carbohydrate, low fat and high protein intake also had a lower risk of CVD. Given that risk associations were evident both for single macronutrients and for combinations with other macronutrients for premature death and for CVD in men, our study has highlighted the potential to target dietary interventions at combinations of macronutrients.

### Macronutrient intake and all-cause mortality

Previous studies in older aged populations have identified a decreased risk of frailty with increased protein intake, with increased frailty then associated with increased risk of death^(6)^. Studies have also identified that levels of protein intake higher than recommended add additional protective effect, particularly when combined with physical activity to build muscle mass^(7)^. While the association between protein intake, frailty and death may provide a mechanism for the association found in the present study, it is important to note that we have not been able to explore this relationship by protein type. This is a limitation as previous studies have identified a negative association between different animal-based sources of protein with mortality^(23)^. Conversely, there is a growing body of evidence investigating the health and environmental benefits of consuming plant-based protein.^(24,25)^ Given the low intakes of fibre in this study, it seems unlikely that consumption of plant-based protein was high; however, this is a speculation that warrants further investigation.

High intakes of carbohydrate were associated with an increased risk of death. The risks and benefits of high carbohydrate and protein intakes, respectively, were reflected when we investigated combinations of dietary factors. Individuals who had diets characterised by low carbohydrate intake, low fat intake, yet high protein intake had a decreased risk of death. It is probable that the benefits of higher protein intake are in part due to the displacement of other macronutrients in the diet.

### Macronutrient intake and CVD and dementia

Dietary risks identified for CVD and dementia were low, and no associations were identified for dietary clusters, with the exception of men with diets characterised by low carbohydrate, low fat and high protein intake having a lower risk of CVD. Individuals with moderate total energy intake had a lower risk of both dementia and CVD. The mechanism for this is unclear and energy intake on its own has not been strongly related to health outcomes^(26)^. For CVD, high sugar intake was associated with an increased risk, yet moderate intake of protein was associated with a lower risk. Previous studies have suggested an association between higher consumption of added sugar with CVD and mortality^(5,26)^. It has been proposed that the mechanism for this is through increased body weight, adverse glycaemic effects and lower intake of other essential nutrients with increasing sugar intake, likely increasing the risk of CVD^(5,27)^. It should be noted that 63 % of the individuals in this study exceeded sugar intake recommendations, and people in the highest third of sugar intake getting 30 % of their total daily energy intake from sugar.

Minimal associations were identified for dementia, with the strongest effects identified for women with moderate sugar intake and high fibre intake having an associated decreased risk of dementia. While we were not able to identify the sources of sugar and fibre in the diet, findings from previous studies showed that compliance to a Mediterranean diet characterised by high consumption of fruits, vegetables, legumes and complex carbohydrates, moderate consumption of fish and olive oil and low consumption of red wine decreased the risk of cognitive impairment and dementia^(28–30)^. In particular, previous studies found that the type of fat consumed was important, with foods richer in polyunsaturated and monounsaturated fats appearing to either improve cognitive function^(30)^ or delay cognitive decline in high-risk groups^(31)^. While in general we did not find an association between type of fat and dementia outcomes, we did identify that women with diets characterised by high polyunsaturated fat intake had a lower relative risk of dementia compared with men. However, this association was attenuated in the model with multiple adjustments. Given the burden of dementia in the UK is predicted to increase by 57 % by 2040^(32)^, there is a need for further research of modifiable lifestyle risk factors for dementia.

### Sex differences in dietary risk

Given previous work that identified sex differences in macronutrient intakes within the UK Biobank population^(14)^, we hypothesised that differences in intake may be associated with corresponding sex differences in our outcomes of interest. While we identified some sex differences in associations between macronutrients, and the outcomes the magnitude of these findings were generally small. Sex differences in the association of individual macronutrients with all-cause mortality were identified. Relative to men, women with the highest third of sugar intake had a lower relative risk of death than those in the lowest third. Conversely, relative to men, women with moderate total fat intake had a higher risk of death than women with a low intake. These observed differences may be due to chance and require confirmation through other studies before firm conclusions can be drawn. However, it is worth noting that sex differences were not evident in the association of dietary clusters with all-cause mortality. For CVD, the individual macronutrient associations that were significant for the population as a whole were only significant for men and not women. This translated into the cluster analysis, where the cluster characterised by low carbohydrate, low fat and high-protein intake cluster was associated with a decreased risk of CVD for men. For women, moderate intakes of sugar and high intakes of fibre were associated with a lower risk of dementia, yet high saturated fat was associated with an increased risk of dementia, and relative to men these findings were significant. While it is plausible that different dietary intakes correspond to differing associations with disease outcomes by sex, the inconsistency between our sex-specific results looking at macronutrients individually and when looking at clusters of macronutrient intakes for all-cause mortality and dementia warrants further investigation.

### Contextualising findings in line with nutrition epidemiology

There is a need to investigate diet quality, for example the source, type and level of processing, in addition to macronutrient intake when looking at the association with disease outcomes. Recent large-scale studies have identified limited associations between individual macronutrient intake with health outcomes^(33–35)^. Yet when the same datasets are used to look at dietary quality (for example, categorisation of ‘healthy’ or ‘unhealthy’ sources of fat and carbohydrate) stronger associations are seen^(33,35)^. Diet quality can also be contextualised in terms of dietary patterns followed. As discussed earlier, a Mediterranean-style diet has been associated with a decreased risk of cognitive decline^(30)^, it has also been associated with a lower incidence of major cardiovascular events in comparison with a low fat diet^(36)^; however, it is important to note that these associations were identified in populations at high risk of vascular diseases at baseline.

Ho et al.^(37)^ previously identified non-linear trends between macronutrient intake with all-cause mortality and CVD in the UK Biobank. The non-linear relationship identified may explain the minimal associations that we found for individual macronutrients with CVD when viewed as thirds of intake. Our findings somewhat echo theirs; however, via cluster analyses, we have further characterised the current dietary patterns of people within the UK Biobank and estimated the risk that intake of different combinations of macronutrients have for disease outcomes. Given the need for dietary interventions that are sustainable and scalable, we suggest that further investigation of diet quality, dietary patterns and associations with disease outcomes are needed.

### Strengths and limitations

A main limitation to the present study is the use of self-reported dietary intake data^(38)^. Dietary self-report is subject to multiple biases, and studies have shown that people randomly and systematically misreport dietary data, for example with people underreporting foods that they consider ‘unhealthy’^(39)^. However, the use of the dietary measure, the Oxford WebQ, has been validated previously^(16)^. The UK Biobank is a volunteer population, and the diet questionnaires were emailed to most participants, with only 20 % of the total population completing two or more diet measures. There is also an indication that the group who completed two or more measures were healthier at baseline than those who completed less than two. While this limits generalisability of our findings, the UK Biobank still provides one of the largest cohorts that has comprehensive diet information and disease outcome information collected prospectively. Additionally, only including individuals with two or more completed measures is more likely to provide a reflection of habitual intake, in comparison with those who only have one measure^(18,26)^. We also excluded individuals who had cardiovascular events or dementia diagnosis between measures, as this may have influenced eating behaviour.

Nutritional epidemiology is complicated by the fact that macronutrients are not consumed in isolation of each other. There are a range of methods that either account for or utilise the compound effects of nutrients. In the present study, we utilised cluster analyses to characterise the study population based on macronutrient intake^(40)^. To the best of our knowledge, this is the largest study to utilise cluster analysis for investigating the association between combinations of macronutrient intake and CVD, dementia and premature death outcomes. In doing this, we have identified combinations of macronutrient intake that could be targeted by food policies. Finally, a number of comparisons were investigated in this study. This means that the risk of type I error will be high, and so significant results should be interpreted with caution. However, in interpreting the results, we mainly focused on effect sizes and their confidence intervals, rather than *P* values.

### Conclusion

In conclusion, the present study identified a range of associations between energy and macronutrient intake and all-cause mortality, CVD and dementia, with some differences by sex identified. Dietary intake was characterised based on cluster analysis, finding that individuals with low carbohydrate, low fat and high protein intake had a lower risk of premature all-cause mortality. Further, men with this dietary cluster also had a lower risk of CVD. Given that associations were evident, both as single macronutrients and in combination with other macronutrients, we suggest the biggest benefit from diet-related policy and interventions would be when combinations of macronutrients are targeted. Since we identified certain sex differences, which require confirmation, we also suggest that associations between diet and disease by sex continue to be investigated.

## Supplementary material

For supplementary material accompanying this paper visit https://doi.org/10.1017/S000711452100266X. click here to view supplementary material
