# Supplementary material for: The association of energy and macronutrient intake with all-cause mortality, cardiovascular disease and dementia: findings from 120 963 women and men in the UK Biobank
Source: Br J Nutr. 2021 Jul 14;127(12):1858–67. doi: 10.1017/S000711452100266X (PMC9244434; doi:10.1017/S000711452100266X)
Supplement: Supplementary file 1 [file S000711452100266Xsup001.docx]

**Online supplementary material**

**Supplementary methods,** cluster analysis

**Supplementary table 1.** Energy and macronutrient intake ranges, by thirds (mean, (min-max))

**Supplementary table 2**. Summary characteristics of participants with two or more dietary assessment measures compared with those without two or more dietary assessment measures, by sex

**Supplementary table 3.** Percentage of the population (n) not meeting the recommended dietary intakes

**Supplementary figure 1.** Macronutrient intake (as a percentage of total energy intake, in thirds) and hazard ratios (HRs) for all-cause mortality (death), subdistribution hazard ratios (SHRs) for cardiovascular disease (CVD) and dementia with 95% confidence intervals (CIs). Models adjusted for age, smoking, sex and Townsend

**Supplementary table 4.** Macronutrient intake (as a percentage of total energy intake, in thirds) and subdistribution hazard ratios (SHRs) for Alzheimer’s disease and vascular dementia with 95% confidence intervals (CIs)

**Supplementary table 5.** Macronutrient intake (as a percentage of total energy intake, in thirds) and hazard ratios (HRs) for all-cause mortality (death)with 95% confidence intervals (CIs), for females, males and the ratio of HRs (RHRs) of females to males.

**Supplementary table 6.** Macronutrient intake (as a percentage of total energy intake, in thirds) and subdistribution hazard ratios (SHRs) for cardiovascular disease (CVD) with 95% confidence intervals (CIs), for females, males and the ratio of HRs (RHRs) of females to males.

**Supplementary table 7.** Macronutrient intake (as a percentage of total energy intake, in thirds) and subdistribution hazard ratios (SHRs) for dementia with 95% confidence intervals (CIs), for females, males and the ratio of HRs (RHRs) of females to males.

**Supplementary table 8.** Exceeding recommended dietary intakes and hazard ratios for death, cardiovascular disease and dementia

**Supplementary figure 2**. Dietary profile of identified clusters, standardised

**Supplementary table 9**. Summary characteristics, by clusters

**Supplementary figure 3.** Hazard ratios (HRs) for outcomes of all-cause mortality (death), and subdistribution hazard ratios (SHRs) for cardiovascular disease (CVD) and dementia with 95% confidence intervals (CIs), from models adjusted for clusters, age, smoking, sex and Townsend. Macronutrient clusters: *up* - low polyunsaturated fat, low protein, *cfP* - low carbohydrate, low fat, high protein, *Cf* – high carbohydrate, low fat, *cF* – low carbohydrate, and high fat, *U*– high polyunsaturated fat.

**Supplementary table 10.** Clusters of dietary intake and hazard ratios (HRs) for all-cause mortality (death)with 95% confidence intervals (CIs), for females, males and the ratio of HRs (RHRs) of females to males. Models adjusted for clusters, age, smoking, and Townsend score (basic adjusted), models adjusted for clusters, age, smoking, height, weight, mean alcohol intake, physical activity (mean total MET), systolic blood pressure, Townsend score, diabetes, lipid lowering medication, anti-hypertensive medication (multiple adjusted).

**Supplementary table 11.** Clusters of dietary intake and subdistribution hazard ratios (SHRs) for cardiovascular disease (CVD) with 95% confidence intervals (CIs), for females, males and the ratio of HRs (RHRs) of females to males. Models adjusted for clusters, age, smoking, and Townsend score (basic adjusted), models adjusted for clusters, age, smoking, height, weight, mean alcohol intake, physical activity (mean total MET), systolic blood pressure, Townsend score, diabetes, lipid lowering medication, anti-hypertensive medication (multiple adjusted).

**Supplementary table 12**. Clusters of dietary intake and subdistribution hazard ratios (SHRs) for dementia with 95% confidence intervals (CIs), for females, males and the ratio of HRs (RHRs) of females to males. Models adjusted for clusters, age, smoking, and Townsend score (basic adjusted), models adjusted for clusters, age, smoking, height, weight, mean alcohol intake, physical activity (mean total MET), systolic blood pressure, Townsend score, diabetes, lipid lowering medication, anti-hypertensive medication (multiple adjusted).

**Supplementary methods, cluster analysis**

Cluster analysis was undertaken using the k-means method, an unsupervised learning algorithm, to partition individuals into k clusters based on patterns in the data, whereby individuals in the same cluster are as similar as possible. The Individuals are represented by cluster centres (centroid) which corresponds to the mean of points assigned to the cluster. The k-means++ algorithm^(1)^ is used from the ClusterR package in R ^(2)^ and is an improved initialisation algorithm to determine the initial cluster centres. A standard initialisation approach, such as the Lloyd algorithm ^(3)^, would arbitrarily choose k centres, whereas the k-means++ algorithm selects the first centre randomly and then for subsequent centres chosen from the remaining data points with probability proportional to its squared distance from the point's closest existing cluster centres. After the centres have been selected via the k-means ++ algorithm, the standard k-means approach is applied that partitions data based on local optima and minimises the total within-cluster variation, defined as the sum of squared distances (sum of squared Euclidean distances) between datapoints and the corresponding cluster centre.

Individuals were clustered based on standardised macronutrients data (% Carbohydrate, % Sugar, % Fibre, % Fat, % Saturated fat, % Polyunsaturated fat, % Protein)

The optimum number of clusters was determined using the ‘elbow’ method based on Bayesian Information Criterion (BIC) calculated over different values of *k* clusters *(k = 2 to 10)*. The elbow (location of a bend) of a function is a point after which the decrease becomes notably smaller. The elbow method to determine the optimum number of clusters is a heuristic approach, since the BIC stop decreasing as much after 5 clusters. Thus the optimum number of clusters was five, based on the elbow method.

Clusters were then described by the characteristics of the macronutrient data. The cluster variable was then inputted as an explanatory variable into separate Cox proportional hazards models for outcomes CVD, Death and Dementia. Two sets of models were fitted to the three outcomes (CVD, Death, Dementia), basic adjusted model adjusting for the cluster variable, age, sex, smoking, Townsend deprivation score, and fully adjusted model additionally adjusting for height, weight, mean alcohol intake, physical activity (mean total MET), systolic blood pressure, diabetes, lipid lowering medication, anti-hypertensive medication .


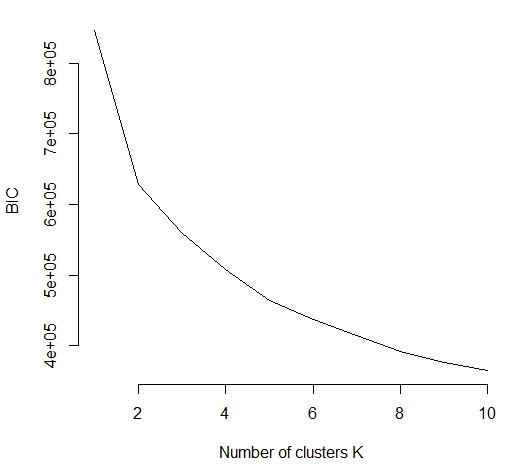


**Supplementary table 1** Energy and macronutrient intake ranges, by thirds (mean (min - max))

| Intake thirds | Energy intake (mean kJ/day) | Carbohydrate (%EI) | Sugar (%EI) | Fibre (%EI) | Fat (%EI) | Saturated Fat (%EI) | Polyunsaturated fat (%EI) | Protein (%EI) |
| --- | --- | --- | --- | --- | --- | --- | --- | --- |
| 1 | 6,572 (1,408 - 7,748) | 40.6 (1.0 - 46.0) | 16.5 (0.3 - 29.3) | 1.0 (0 - 1.3) | 26.2 (3.5 -30.1) | 9.3 (0.9 - 11.2) | 4.0 (0.4 - 5.0) | 13.0 (3.5 - 14.6) |
| 2 | 8,601 (7,748 - 9,508) | 49.1 (46.0 - 52.2) | 22.9 (20.3 - 25.6) | 1.5 (1.3 - 1.7) | 32.5 (30.1 - 35.0) | 12.4 (11.2 - 13.6) | 5.8 (5.0 - 6.7) | 15.8 (14.6 - 17.1) |
| 3 | 11,286 (9,509 - 19,786) | 56.9 (52.2 - 89.3) | 30.2 (25.6 - 71.1) | 2.0 (1.7 - 6.5) | 38.8 (35.0 - 66.7) | 15.7 (13.6 - 27.8) | 8.1 (6.7 - 19.1) | 19.5 (17.1 - 50.0) |

*%EI= percentage of energy intake

**Supplementary table 2**. Summary characteristics of participants with two or more dietary assessment measures compared with those without two or more dietary assessment measures, by sex

| **Characteristics** | **Women** | | **Men** | |
| --- | --- | --- | --- | --- |
|  | Completed ≥2 24hr diet recalls | Completed <2 24hr diet recalls | Completed ≥2 24hr diet recalls | Completed <2 24hr diet recalls |
| n | 68,927 | 204,450 | 52,036 | 177,079 |
| Age, years (SD) | 55.5 (7.7) | 56.6 (8.1) | 56.5 (7.9) | 56.8 (8.3) |
| Ethnicity, white (%) | 66,510 (96.8) | 190,921 (93.4) | 50,245 (97.0) | 165,007 (93.2) |
| Socioeconomic status quintiles (%) |  |  |  |  |
| 1^st^ Least deprived | 26,880 (39.0) | 74,118 (36.3) | 21,455 (41.3) | 63,423 (35.8) |
| 2^nd^ | 14,728 (21.4) | 41,862 (20.5) | 10,823 (20.8) | 35,357 (20.0) |
| 3^rd^ | 10,693 (15.5) | 30,553 (14.9) | 7,818 (15.0) | 25,517 (14.4) |
| 4^th^ | 9,214 (13.4) | 27,695 (13.5) | 6,450 (12.4) | 24,006 (13.6) |
| 5^th^ Most deprived | 7,330 (10.6) | 29,977 (14.7) | 5,430 (10.4) | 28,540 (16.1) |
| Smoking status, never smoked (%) | 42,195 (61.3) | 119,857 (58.6) | 27,745 (53.4) | 83,720 (47.3) |
| BMI, kg/m^2^ (SD) | 26.2 (4.9) | 27.4 (5.3) | 27.2 (4.0) | 280.0 (4.3) |
| Overweight or obese (%) | 36,109 (52.5) | 128,070 (62.6) | 35,876 (69.1) | 134,300 (75.8) |
| Weight, kg (SD) | 70.2 (13.6) | 71.9 (14.3) | 85.0 (13.7) | 86.2 (14.5) |
| Height, cm (SD) | 163.6 (6.2) | 162.1 (6.3) | 176.8 (6.7) | 175.3 (6.9) |
| Low physical activity (%)^1^ | 12,194 (18.8) | 36,982 (18.1) | 9,447 (18.9) | 30,575 (17.3) |
| Systolic blood pressure (mmHg) | 133.7 (18.7) | 135.9 (19.4) | 140.4 (16.9) | 141.1 (17.6) |
| Diastolic blood pressure (mmHg) | 80.2 (9.8) | 80.9 (10.0) | 83.9 (9.7) | 84.1 (10.1) |
| Blood pressure categories (%)^2^ |  |  |  |  |
| Normal | 15,505 (22.5) | 39,827 (19.5) | 4,473 (8.6) | 15,557 (8.8) |
| Elevated | 9,709 (14.1) | 25,866 (12.7) | 6,314 (12.1) | 19,711 (11.1) |
| Stage 1 hypertension | 18,559 (27.0) | 53,507 (26.2) | 14,813 (28.5) | 47,892 (27.0) |
| Stage 2 hypertension | 25,082 (36.4) | 84,551 (41.4) | 26,417 (50.8) | 93,384 (52.7) |

**Supplementary table 3**. Percentage of the population (n) not meeting the recommended dietary intakes^1^

|  | Overall | Females | Males |
| --- | --- | --- | --- |
| Energy intake (EI) |  |  |  |
| ≥10,460kJ for men, ≥8,363kJ for women | 38.3 (46,324) | 43.5 (29,960) | 31.5 (16,364) |
|  |  |  |  |
| Carbohydrate intake |  |  |  |
| Total carbohydrate <50% EI | 54.8 (66,287) | 52.4 (36,145) | 57.9 (30,142) |
| Sugar ≥120g for men, ≥90g for women | 63.0 (76,145) | 72.5 (49,984) | 50.3 (26,161) |
| Fibre <30g | 97.7 (118,168) | 98.0 (67,565) | 97.3 (50,603) |
|  |  |  |  |
| Fat intake |  |  |  |
| Total fat ≥35% EI | 33.3 (40,267) | 34.57 (23,827) | 31.6 (16,440) |
| Saturated fat ≥11% EI | 68.5 (82,897) | 69.1 (47,652) | 67.7 (35,245) |
| Polyunsaturated fat <6% or >11% EI | 55.0 (66,572) | 53.6 (36,918) | 57.0 (29,654) |
|  |  |  |  |
| Protein intake |  |  |  |
| Protein intake <0.75g per kg body weight | 12.0 (14,545) | 9.9 (6,803) | 14.9 (7,742) |
|  |  |  |  |

^1^UK dietary recommendations ^(4)^


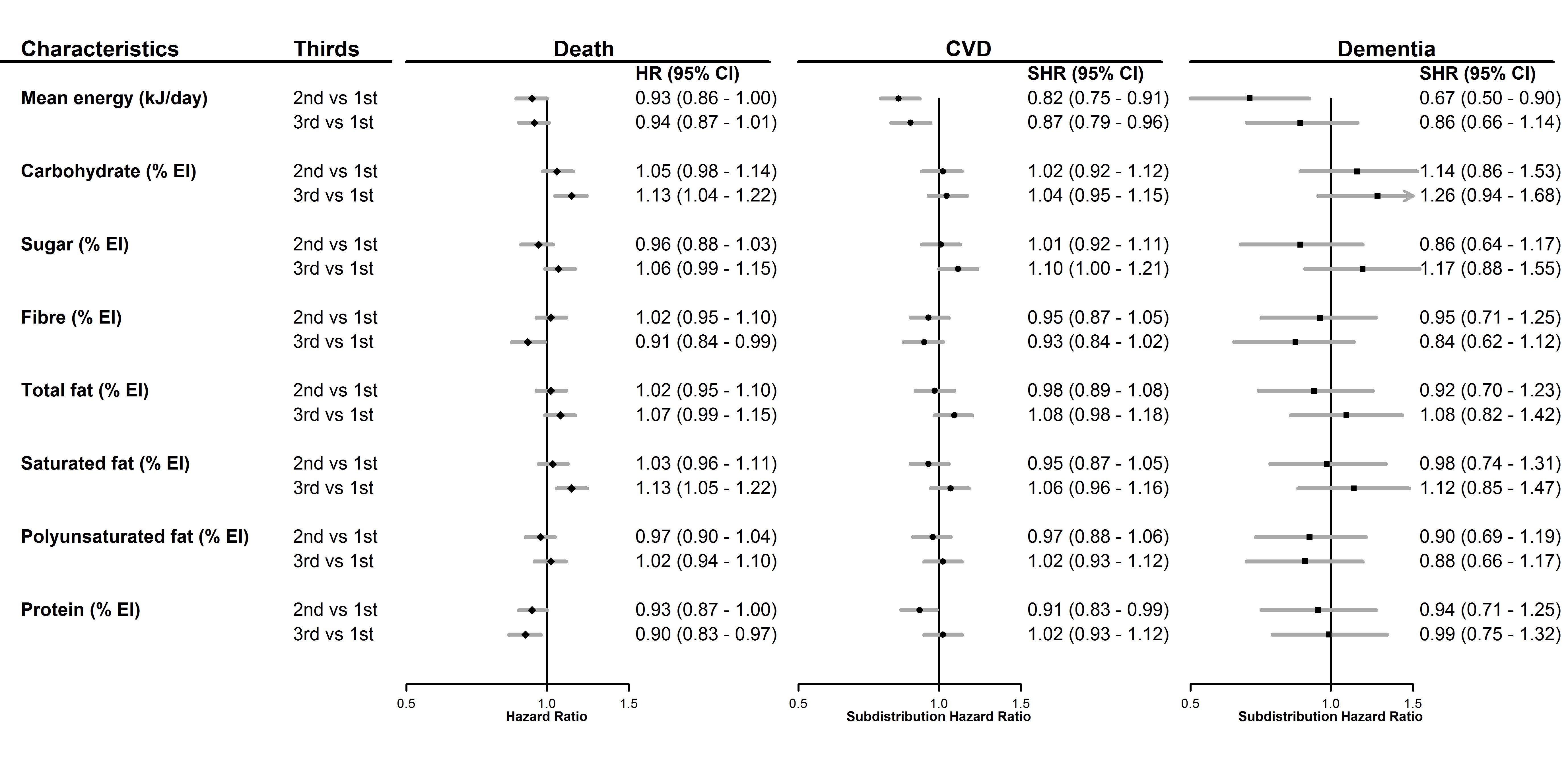


**Supplementary figure 1**. Macronutrient intake (as a percentage of total energy intake (EI), in thirds) and hazard ratios (HRs) for all-cause mortality (death), subdistribution hazard ratios (SHRs) for cardiovascular disease (CVD) and dementia with 95% confidence intervals (CIs).

Models adjusted for age, smoking, sex and Townsend score

**Supplementary table 4.** Macronutrient intake (as a percentage of total energy intake, in thirds) and subdistribution hazard ratios (SHRs) for Alzheimer’s disease and vascular dementia with 95% confidence intervals (CIs)

| **Intake of macronutrients (%EI)** | **Alzheimer’s disease SHR** | **Vascular dementia SHR** |
| --- | --- | --- |
| **Mean energy intake (kJ/day)** |  |  |
| 1 |  |  |
| 2 | 0.64 (0.38, 1.07) | 0.86 (0.42, 1.76) |
| 3 | 0.63 (0.37, 1.09) | 1.42 (0.72, 2.79) |
| **Carbohydrate intake (% EI)** |  |  |
| 1 |  |  |
| 2 | 1.19 (0.68, 2.09) | 0.82 (0.40, 1.68) |
| 3 | 0.90 (0.48, 1.67) | 0.94 (0.45, 1.96) |
| **Sugar intake (% EI)** |  |  |
| 1 |  |  |
| 2 | 0.69 (0.40, 1.20) | 0.69 (0.35, 1.37) |
| 3 | 0.86 (0.50, 1.46) | 0.78 (0.40, 1.54) |
| **Fibre intake (% EI)** |  |  |
| 1 |  |  |
| 2 | 1.05 (0.61, 1.81) | 0.68 (0.34, 1.35) |
| 3 | 0.98 (0.56, 1.71) | 0.86 (0.44, 1.66) |
| **Total fat intake (% EI)** |  |  |
| 1 |  |  |
| 2 | 0.85 (0.50, 1.45) | 1.16 (0.61, 2.23) |
| 3 | 1.02 (0.61, 1.73) | 1.07 (0.54, 2.12) |
| **Saturated fat intake (% EI)** |  |  |
| 1 |  |  |
| 2 | 0.92 (0.54, 1.57) | 0.85 (0.45, 1.63) |
| 3 | 1.04 (0.62, 1.75) | 0.85 (0.44, 1.63) |
| **Polyunsaturated fat intake (% EI)** |  |  |
| 1 |  |  |
| 2 | 0.98 (0.58, 1.65) | 1.09 (0.55, 2.16) |
| 3 | 1.04 (0.61, 1.75) | 1.42 (0.74, 2.74) |
| **Protein intake (% EI)** |  |  |
| 1 |  |  |
| 2 | 1.07 (0.64, 1.79) | 1.03 (0.55, 1.91) |
| 3 | 1.02 (0.59, 1.76) | 0.77 (0.38, 1.56) |
|  |  |  |

*% EI, percentage of energy intake

** Adjusted for age, sex, smoking, height, weight, mean alcohol intake, physical activity (mean total MET), systolic blood pressure, Townsend score, diabetes, lipid lowering medication, anti-hypertensive medication

**Supplementary table 5.** Macronutrient intake (as a percentage of total energy intake, in thirds) and hazard ratios (HRs) for all-cause mortality (death) with 95% confidence intervals (CIs), for females and males and the female to male ratio of HRs (RHRs).

| **Intake of macronutrients (%EI)** | **Female HR** | **Male HR** | **Female to male RHR** |
| --- | --- | --- | --- |
| **Mean energy intake (kJ/day)** |  |  |  |
| 1 |  |  |  |
| 2 | 0.99 (0.89, 1.11) | 0.96 (0.85, 1.08) | 1.03 (0.88, 1.21) |
| 3 | 1.06 (0.94, 1.21) | 0.97 (0.86, 1.09) | 1.09 (0.92, 1.30) |
| **Carbohydrate intake (% EI)** |  |  |  |
| 1 |  |  |  |
| 2 | 1.08 (0.95, 1.22) | 1.03 (0.92, 1.15) | 1.05 (0.89, 1.24) |
| 3 | 1.17 (1.02, 1.34) | 1.08 (0.95, 1.22) | 1.08 (0.90, 1.30) |
| **Sugar intake (% EI)** |  |  |  |
| 1 |  |  |  |
| 2 | 0.88 (0.77, 1.00) | 1.03 (0.93, 1.15) | 0.85 (0.72, 1.01) |
| 3 | 0.95 (0.84, 1.08) | 1.17 (1.05, 1.31) | 0.81 (0.69, 0.96) |
| **Fibre intake (% EI)** |  |  |  |
| 1 |  |  |  |
| 2 | 1.03 (0.91, 1.17) | 1.01 (0.91, 1.11) | 1.02 (0.87, 1.20) |
| 3 | 0.93 (0.82, 1.06) | 0.89 (0.79, 0.99) | 1.04 (0.88, 1.24) |
| **Total fat intake (% EI)** |  |  |  |
| 1 |  |  |  |
| 2 | 1.12 (1.00, 1.27) | 0.93 (0.84, 1.03) | 1.20 (1.03, 1.41) |
| 3 | 1.07 (0.95, 1.21) | 0.96 (0.86, 1.07) | 1.11 (0.95, 1.31) |
| **Saturated fat intake (% EI)** |  |  |  |
| 1 |  |  |  |
| 2 | 0.98 (0.87, 1.11) | 1.02 (0.92, 1.13) | 0.96 (0.82, 1.13) |
| 3 | 1.12 (0.99, 1.26) | 1.02 (0.91, 1.14) | 1.10 (0.93, 1.30) |
| **Polyunsaturated fat intake (% EI)** |  |  |  |
| 1 |  |  |  |
| 2 | 0.95 (0.85, 1.07) | 0.98 (0.88, 1.08) | 0.97 (0.83, 1.13) |
| 3 | 0.98 (0.87, 1.10) | 1.01 (0.91, 1.12) | 0.97 (0.83, 1.13) |
| **Protein intake (% EI)** |  |  |  |
| 1 |  |  |  |
| 2 | 0.89 (0.79, 1.00) | 0.94 (0.85, 1.04) | 0.94 (0.81, 1.11) |
| 3 | 0.78 (0.69, 0.88) | 0.86 (0.77, 0.96) | 0.91 (0.77, 1.07) |
|  |  |  |  |

*% EI, percentage of energy intake

** Adjusted for age, smoking, height, weight, mean alcohol intake, physical activity (mean total MET), systolic blood pressure, Townsend score, diabetes, lipid lowering medication, anti-hypertensive medication

**Supplementary table 6.** Macronutrient intake (as a percentage of total energy intake, in thirds) and subdistribution hazard ratios (SHRs) for cardiovascular disease (CVD) with 95% confidence intervals (CIs), for females and males and the female to male ratio of SHRs (RSHRs).

| **Intake of macronutrients (%EI)** | **Female SHR** | **Male SHR** | **Female to male RSHR** |
| --- | --- | --- | --- |
| **Mean energy intake (kJ/day)** |  |  |  |
| 1 |  |  |  |
| 2 | 0.94 (0.81, 1.09) | 0.83 (0.72, 0.95) | 1.13 (0.92, 1.39) |
| 3 | 0.96 (0.80, 1.14) | 0.94 (0.83, 1.07) | 1.02 (0.82, 1.27) |
| **Carbohydrate intake (% EI)** |  |  |  |
| 1 |  |  |  |
| 2 | 1.08 (0.91, 1.28) | 1.00 (0.88, 1.13) | 1.08 (0.87, 1.33) |
| 3 | 1.05 (0.87, 1.26) | 1.01 (0.88, 1.17) | 1.04 (0.82, 1.31) |
| **Sugar intake (% EI)** |  |  |  |
| 1 |  |  |  |
| 2 | 1.02 (0.85, 1.21) | 1.07 (0.95, 1.21) | 0.95 (0.77, 1.18) |
| 3 | 1.07 (0.90, 1.27) | 1.17 (1.02, 1.33) | 0.91 (0.74, 1.14) |
| **Fibre intake (% EI)** |  |  |  |
| 1 |  |  |  |
| 2 | 0.88 (0.74, 1.05) | 0.99 (0.88, 1.11) | 0.89 (0.72, 1.10) |
| 3 | 0.94 (0.79, 1.11) | 0.87 (0.76, 0.99) | 1.08 (0.87, 1.34) |
| **Total fat intake (% EI)** |  |  |  |
| 1 |  |  |  |
| 2 | 1.07 (0.91, 1.26) | 0.94 (0.83, 1.06) | 1.14 (0.93, 1.40) |
| 3 | 1.05 (0.89, 1.24) | 1.05 (0.92, 1.18) | 1.00 (0.81, 1.23) |
| **Saturated fat intake (% EI)** |  |  |  |
| 1 |  |  |  |
| 2 | 0.93 (0.79, 1.10) | 0.95 (0.84, 1.08) | 0.98 (0.80, 1.21) |
| 3 | 1.00 (0.85, 1.17) | 1.06 (0.94, 1.20) | 0.94 (0.77, 1.15) |
| **Polyunsaturated fat intake (% EI)** |  |  |  |
| 1 |  |  |  |
| 2 | 0.85 (0.72, 1.01) | 1.01 (0.90, 1.14) | 0.84 (0.68, 1.03) |
| 3 | 1.03 (0.88, 1.21) | 0.96 (0.85, 1.09) | 1.07 (0.88, 1.31) |
| **Protein intake (% EI)** |  |  |  |
| 1 |  |  |  |
| 2 | 0.89 (0.75, 1.05) | 0.87 (0.77, 0.98) | 1.02 (0.83, 1.26) |
| 3 | 0.96 (0.81, 1.13) | 0.87 (0.77, 0.99) | 1.10 (0.90, 1.36) |
|  |  |  |  |

*% EI, percentage of energy intake

** Adjusted for age, smoking, height, weight, mean alcohol intake, physical activity (mean total MET), systolic blood pressure, Townsend score, diabetes, lipid lowering medication, anti-hypertensive medication

**Supplementary table 7.** Macronutrient intake (as a percentage of total energy intake, in thirds) and subdistribution hazard ratios (SHRs) for dementia with 95% confidence intervals (CIs), for females and males and the female to male ratio of SHRs (RSHRs).

| **Intake of macronutrients (%EI)** | **Female SHR** | **Male SHR** | **Female to male RSHR** |
| --- | --- | --- | --- |
| **Mean energy intake (kJ/day)** |  |  |  |
| 1 |  |  |  |
| 2 | 0.75 (0.50, 1.13) | 0.69 (0.43, 1.12) | 1.19 (0.58, 2.04) |
| 3 | 0.72 (0.44, 1.18) | 1.05 (0.68, 1.62) | 0.69 (0.36, 1.32) |
| **Carbohydrate intake (% EI)** |  |  |  |
| 1 |  |  |  |
| 2 | 0.82 (0.52, 1.31) | 1.27 (0.82, 1.96) | 0.65 (0.34, 1.22) |
| 3 | 0.76 (0.46, 1.24) | 1.38 (0.85, 2.25) | 0.55 (0.27, 1.10) |
| **Sugar intake (% EI)** |  |  |  |
| 1 |  |  |  |
| 2 | 0.51 (0.30, 0.85) | 1.27 (0.85, 1.89) | 0.40 (0.21, 0.77) |
| 3 | 0.90 (0.58, 1.40) | 1.18 (0.76, 1.83) | 0.76 (0.41, 1.42) |
| **Fibre intake (% EI)** |  |  |  |
| 1 |  |  |  |
| 2 | 0.70 (0.45, 1.09) | 1.12 (0.75, 1.67) | 0.62 (0.34, 1.13) |
| 3 | 0.57 (0.37, 0.88) | 1.10 (0.71, 1.69) | 0.52 (0.28, 0.96) |
| **Total fat intake (% EI)** |  |  |  |
| 1 |  |  |  |
| 2 | 0.94 (0.60, 1.47) | 0.86 (0.58, 1.26) | 1.09 (0.60, 1.98) |
| 3 | 1.17 (0.76, 1.79) | 0.86 (0.57, 1.30) | 1.36 (0.75, 2.47) |
| **Saturated fat intake (% EI)** |  |  |  |
| 1 |  |  |  |
| 2 | 1.39 (0.86, 2.25) | 0.74 (0.50, 1.10) | 1.88 (1.01, 3.50) |
| 3 | 1.69 (1.06, 2.68) | 0.68 (0.45, 1.03) | 2.49 (1.33, 4.63) |
| **Polyunsaturated fat intake (% EI)** |  |  |  |
| 1 |  |  |  |
| 2 | 0.82 (0.54, 1.26) | 1.04 (0.71, 1.52) | 0.79 (0.45, 1.39) |
| 3 | 0.75 (0.49, 1.16) | 0.90 (0.60, 1.36) | 0.83 (0.46, 1.51) |
| **Protein intake (% EI)** |  |  |  |
| 1 |  |  |  |
| 2 | 0.85 (0.54, 1.34) | 1.05 (0.71, 1.55) | 0.81 (0.44, 1.47) |
| 3 | 0.89 (0.57, 1.39) | 1.03 (0.66, 1.57) | 0.87 (0.47, 1.62) |
|  |  |  |  |

*% EI, percentage of energy intake

** Adjusted for age, smoking, height, weight, mean alcohol intake, physical activity (mean total MET), systolic blood pressure, Townsend score, diabetes, lipid lowering medication, anti-hypertensive medication

**Supplementary table 8.** Hazard ratios (95%CI) for death, cardiovascular disease (CVD) and dementia for exceeding vs not recommended dietary intakes^1^

|  | **Death, all cause** | **CVD** | **Dementia** |
| --- | --- | --- | --- |
| Energy intake (EI) |  |  |  |
| ≥8,363kJ for women, ≥10,460kJ for men | 0.99 (0.93, 1.07) | 1.01 (0.93, 1.10) | 1.03 (0.80, 1.33) |
|  |  |  |  |
| Carbohydrate intake |  |  |  |
| Total carbohydrate <50% EI | 0.93 (0.86, 0.99) | 0.94 (0.86, 1.02) | 0.98 (0.75, 1.28) |
| Sugar ≥90g for women, ≥120g for men | 1.01 (0.95, 1.09) | 1.04 (0.96, 1.13) | 1.15 (0.88, 1.51) |
| Fibre <30g | 0.93 (0.76, 1.14) | 1.09 (0.84, 1.42) | 0.56 (0.31, 1.01) |
|  |  |  |  |
| Fat intake |  |  |  |
| Total fat ≥35% EI | 1.00 (0.93, 1.07) | 1.06 (0.97, 1.15) | 1.06 (0.81, 1.37) |
| Saturated fat ≥11% EI | 1.03 (0.96, 1.11) | 0.99 (0.90, 1.08) | 0.94 (0.73, 1.23) |
| Polyunsaturated fat <6% or >11% EI | 0.97 (0.91, 1.04) | 0.98 (0.90, 1.06) | 1.23 (0.96, 1.57) |
|  |  |  |  |
| Protein intake |  |  |  |
| Protein intake <0.75g per kg body weight | 1.19 (1.08, 1.30) | 1.17 (1.04, 1.31) | 1.23 (0.83, 1.81) |
|  |  |  |  |

^1^UK dietary recommendations ^(4)^

Models adjusted for age, smoking, sex, height, weight, mean alcohol intake, physical activity (mean total MET), Townsend score, systolic blood pressure, anti-hypertensive medication, diabetes, lipid lowering medication.

**Supplementary table 9.** Summary characteristics (mean (SD), unless stated) and incidence rates per 10,000 person years (for CVD, Death and Dementia) by clusters

| **Cluster** | ***up*** | ***cfP*** | ***Cf*** | ***cF*** | ***U*** |
| --- | --- | --- | --- | --- | --- |
| *n* | 30231 | 22700 | 22215 | 23668 | 22149 |
| *Variable* |  |  |  |  |  |
| Sex (% Male) | 46.7 | 47.0 | 35.3 | 45.6 | 38.8 |
| Age (years) | 56.1 (7.9) | 55.7 (7.8) | 56.6 (7.6) | 55.3 (7.9) | 55.8 (7.8) |
| Smoking (% never smoking) | 60.0 | 52.2 | 62.6 | 53.8 | 60.1 |
| Townsend | -1.65 (2.84) | -1.67 (2.83) | -1.67 (2.84) | -1.53 (2.86) | -1.70 (2.80) |
|  |  |  |  |  |  |
| Height (cm) | 170.1 (9.2) | 169.5 (8.9) | 167.8 (8.9) | 170.0 (9.2) | 168.6 (9.0) |
| Weight (Kg) | 76.5 (15.2) | 78.3 (15.5) | 73.9 (14.7) | 79 (16.4) | 74.9 (14.9) |
| Metabolic equivalents | 2564.32 (3132.22) | 2416.96 (2839.10) | 2813.39 (3181.61) | 2373.45 (2960.96) | 2546.34 (2983.40) |
| SBP (mmHg) | 136.2 (18.1) | 138.1 (18.3) | 136.6 (18.6) | 136 (17.9) | 136 (18.3) |
| Diabetes (%) | 2.4 | 4.0 | 2.8 | 4.3 | 3.9 |
| Lipid lowering medication (%) | 8.5 | 11.5 | 9.4 | 9.8 | 9.1 |
| Anti-hypertensives (%) | 10.5 | 13.6 | 11.5 | 11.7 | 11.4 |
|  |  |  |  |  |  |
| Energy (kJ/day) | 9461 (2329) | 8174 (1978) | 7951 (1952) | 9245 (2301) | 9024 (2108) |
|  |  |  |  |  |  |
| *Incidence rates per 10,000 person years (95%CI)* |  |  |  |  |  |
| CVD | 20.86 (19.31, 22.41) | 19.40 (17.67, 21.13) | 18.76 (17.04, 20.48) | 19.89 (18.18, 21.61) | 19.06 (17.33, 20.79) |
| Death | 32.63 (30.69, 34.56) | 27.88 (25.82, 29.45) | 29.35 (27.21, 31.49) | 31.16 (29.02, 33.30) | 29.01 (26.88, 31.15) |
| Dementia | 2.27 (1.76, 2.78) | 1.84 (1.31, 2.37) | 2.57 (1.93, 3.20) | 2.25 (1.68, 2.83) | 1.96 (1.41, 2.51) |

Macronutrient clusters: *up* - low polyunsaturated fat, low protein, *cfP* - low carbohydrate, low fat, high protein, *Cf* – high carbohydrate, low fat, *cF* – low carbohydrate, and high fat, *U*– high polyunsaturated fat


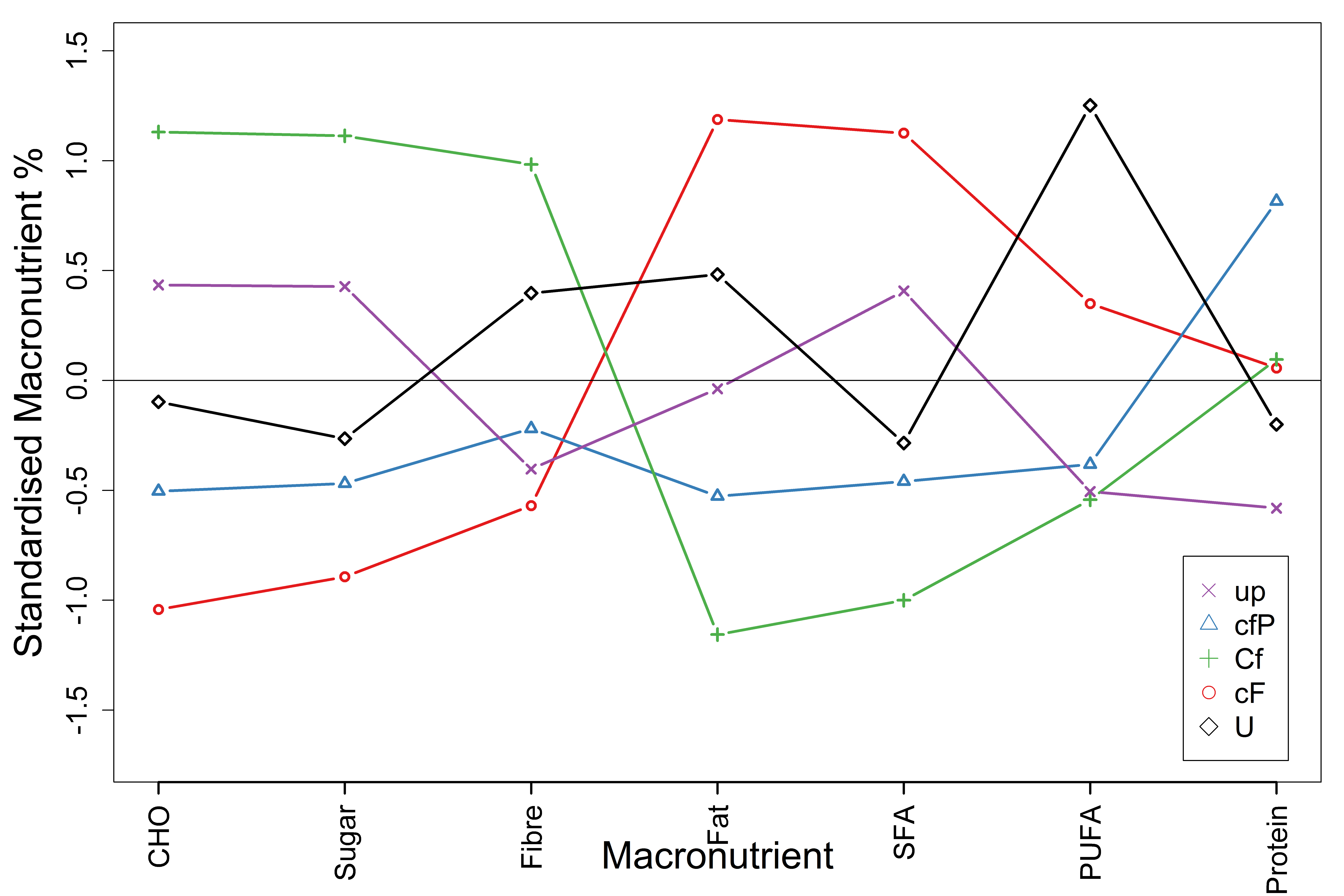


**Supplementary figure 2.** Dietary profile of identified clusters, by (standardised) macronutrient %

Macronutrient clusters: *up* - low polyunsaturated fat, low protein, *cfP* - low carbohydrate, low fat, high protein, *Cf* – high carbohydrate, low fat, *cF* – low carbohydrate, and high fat, *U*– high polyunsaturated fat


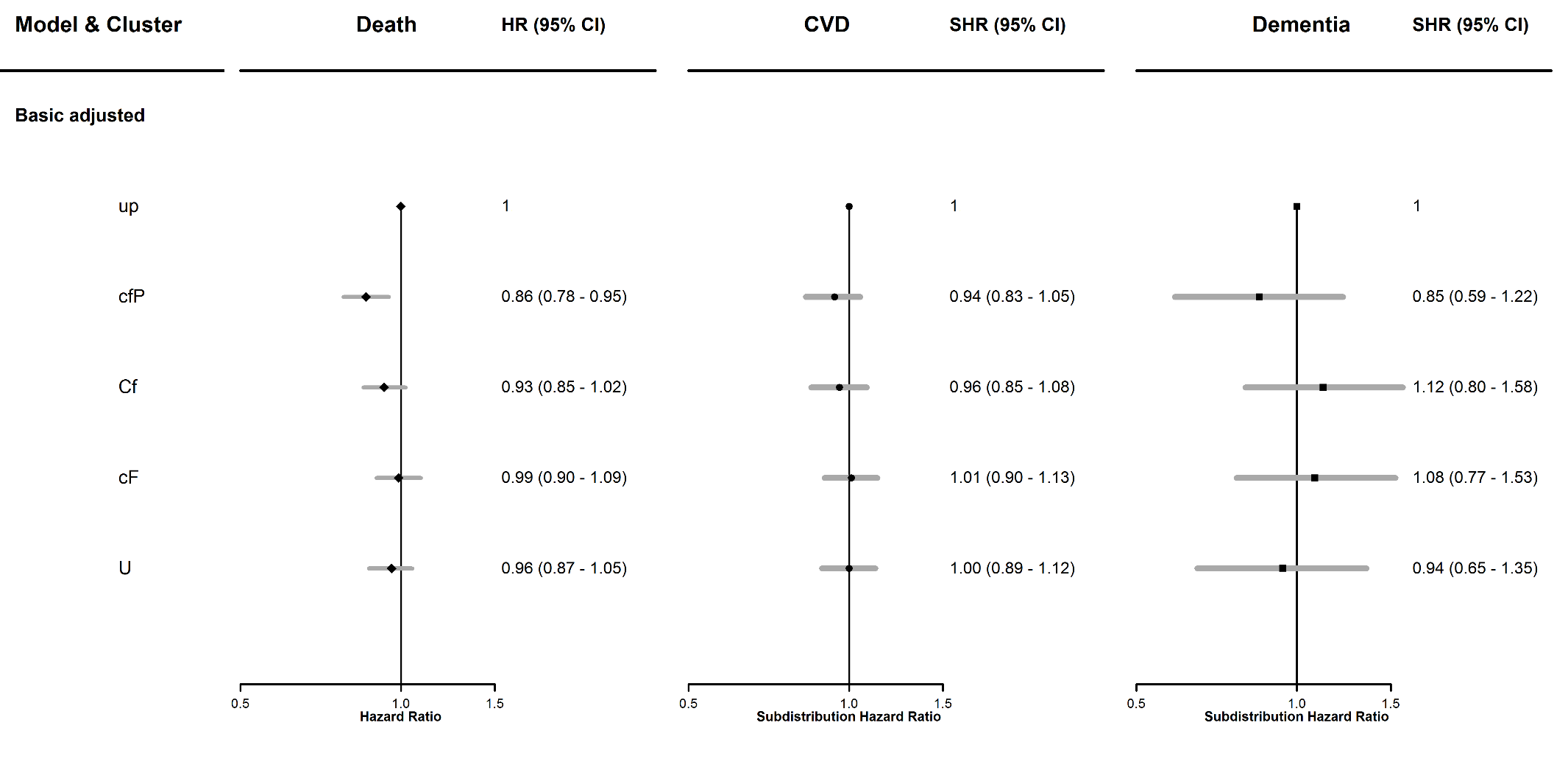


**Supplementary figure 3.** Hazard ratios (HRs) for outcomes of all-cause mortality (death), and subdistribution hazard ratios (SHRs) for cardiovascular disease (CVD) and dementia with 95% confidence intervals (CIs), from models adjusted for clusters, age, smoking, sex and Townsend score. Macronutrient clusters: *up* - low polyunsaturated fat, low protein, *cfP* - low carbohydrate, low fat, high protein, *Cf* – high carbohydrate, low fat, *cF* – low carbohydrate, and high fat, *U*– high polyunsaturated fat.

**Supplementary table 10.** Clusters of dietary intake and hazard ratios (HRs) for all-cause mortality (death) with 95% confidence intervals (CIs), for females and males and the female to male ratio of HRs (RHRs). Models adjusted for clusters, age, smoking, and Townsend score (basic adjusted), models adjusted for clusters, age, smoking, height, weight, mean alcohol intake, physical activity (mean total MET), systolic blood pressure, Townsend score, diabetes, lipid lowering medication, anti-hypertensive medication (multiple adjusted).

| **Cluster** | **Females, HR (95% CI)** | **Males, HR (95% CI)** | **Female to male RHR** |
| --- | --- | --- | --- |
| **Basic adjusted** |  |  |  |
| *up* | ref | ref | ref |
| *cfP* | 0.80 (0.68, 0.93) | 0.90 (0.80, 1.02) | 0.88 (0.73, 1.07) |
| *Cf* | 0.93 (0.81, 1.07) | 0.93 (0.81, 1.06) | 1.01 (0.83, 1.22) |
| *cF* | 0.99 (0.86, 1.14) | 0.99 (0.88, 1.12) | 1.00 (0.84, 1.21) |
| *U* | 0.95 (0.83, 1.09) | 0.96 (0.84, 1.09) | 0.99 (0.82, 1.20) |
| **Multiple adjusted** |  |  |  |
| *up* | ref | ref | ref |
| *cfP* | 0.82 (0.70, 0.96) | 0.86 (0.75, 0.98) | 0.95 (0.77, 1.18) |
| *Cf* | 0.91 (0.79, 1.05) | 0.95 (0.83, 1.09) | 0.96 (0.79, 1.17) |
| *cF* | 0.98 (0.84, 1.13) | 0.94 (0.83, 1.07) | 1.04 (0.85, 1.26) |
| *U* | 0.95 (0.83, 1.10) | 0.95 (0.83, 1.08) | 1.01 (0.83, 1.23) |

Macronutrient clusters: *up* - low polyunsaturated fat, low protein, *cfP* - low carbohydrate, low fat, high protein, *Cf* – high carbohydrate, low fat, *cF* – low carbohydrate, and high fat, *U*– high polyunsaturated fat

**Supplementary table 11.** Clusters of dietary intake and subdistribution hazard ratios (SHRs) for cardiovascular disease (CVD) with 95% confidence intervals (CIs), for females and males and the female to male ratio of SHRs (RSHRs) of females to males. Models adjusted for clusters, age, smoking, and Townsend score (basic adjusted), models adjusted for clusters, age, smoking, height, weight, mean alcohol intake, physical activity (mean total MET), systolic blood pressure, Townsend score, diabetes, lipid lowering medication, anti-hypertensive medication (multiple adjusted).

| **Cluster** | **Females, SHR (95% CI)** | **Males, SHR (95% CI)** | **Female to male RSHR** |
| --- | --- | --- | --- |
| **Basic adjusted** |  |  |  |
| *up* | ref | ref | ref |
| *cfP* | 1.06 (0.87, 1.29) | 0.88 (0.76, 1.01) | 1.21 (0.95, 1.55) |
| *Cf* | 0.92 (0.76, 1.11) | 1.00 (0.86, 1.16) | 0.92 (0.72, 1.17) |
| *cF* | 0.91 (0.74, 1.11) | 1.05 (0.92, 1.21) | 0.86 (0.67, 1.10) |
| *U* | 1.08 (0.89, 1.30) | 0.94 (0.81, 1.10) | 1.14 (0.90, 1.46) |
| **Multiple adjusted** |  |  |  |
| *up* | ref | ref | ref |
| *cfP* | 1.02 (0.83, 1.26) | 0.83 (0.71, 0.97) | 1.23 (0.95, 1.59) |
| *Cf* | 0.87 (0.72, 1.06) | 0.97 (0.83, 1.14) | 0.90 (0.70, 1.15) |
| *cF* | 0.83 (0.67, 1.03) | 1.01 (0.87, 1.17) | 0.82 (0.63, 1.07) |
| *U* | 1.02 (0.84, 1.24) | 0.93 (0.79, 1.09) | 1.10 (0.85, 1.41) |

Macronutrient clusters: *up* - low polyunsaturated fat, low protein, *cfP* - low carbohydrate, low fat, high protein, *Cf* – high carbohydrate, low fat, *cF* – low carbohydrate, and high fat, *U*– high polyunsaturated fat

**Supplementary table 12.** Clusters of dietary intake and subdistribution hazard ratios (SHRs) for dementia with 95% confidence intervals (CIs), for females and males and the female to male ratio of SHRs (RSHRs). Models adjusted for clusters, age, smoking, and Townsend score (basic adjusted), models adjusted for clusters, age, smoking, height, weight, mean alcohol intake, physical activity (mean total MET), systolic blood pressure, Townsend score, diabetes, lipid lowering medication, anti-hypertensive medication (multiple adjusted).

| **Cluster** | **Females, SHR (95% CI)** | **Males, SHR (95% CI)** | **Female to male RSHR** |
| --- | --- | --- | --- |
| **Basic adjusted** |  |  |  |
| *up* | ref | ref | ref |
| *cfP* | 0.72 (0.42, 1.23) | 0.98 (0.59, 1.63) | 0.73 (0.35, 1.53) |
| *Cf* | 0.84 (0.52, 1.34) | 1.51 (0.94, 2.45) | 0.55 (0.28, 1.08) |
| *cF* | 1.08 (0.68, 1.73) | 1.07 (0.65, 1.77) | 1.01 (0.51, 2.00) |
| *U* | 0.57 (0.33, 1.00) | 1.43 (0.88, 2.32) | 0.40 (0.19, 0.84) |
| **Multiple adjusted** |  |  |  |
| *up* | ref | ref | ref |
| *cfP* | 0.76 (0.41, 1.38) | 1.06 (0.63, 1.78) | 0.72 (0.32, 1.59) |
| *Cf* | 0.82 (0.49, 1.35) | 1.43 (0.87, 2.34) | 0.57 (0.28, 1.16) |
| *cF* | 1.17 (0.71, 1.95) | 1.04 (0.62, 1.77) | 1.12 (0.54, 2.33) |
| *U* | 0.61 (0.34, 1.08) | 1.25 (0.75, 2.08) | 0.49 (0.23, 1.05) |

Macronutrient clusters: *up* - low polyunsaturated fat, low protein, *cfP* - low carbohydrate, low fat, high protein, *Cf* – high carbohydrate, low fat, *cF* – low carbohydrate, and high fat, *U*– high polyunsaturated fat

References

1. Arthur D, Vassilvitskii S. k-means++: The Advantages of Careful Seeding, SODA’07 Proceedings of the eighteenth annual ACM-SIAM symposium on Discrete algorithms, New Orleans, Louisiana, 2007. URL <https://theory.stanford.edu/~sergei/papers/kMeansPP-soda.pdf>.

2. Lampros Mouselimis. ClusterR: Gaussian Mixture Models, K-Means, Mini-Batch-Kmeans, K-Medoids and Affinity Propagation Clustering. R package version 1.2.2. 2020. Available from: <https://CRAN.R-project.org/package=ClusterR>.

3. Lloyd S. Least squares quantization in PCM. IEEE transactions on information theory. 1982;28(2):129-37.

4. Public Health England. Government Dietary Recommendations Public Health England 2016. <https://assets.publishing.service.gov.uk/government/uploads/system/uploads/attachment_data/file/618167/government_dietary_recommendations.pdf>
